# Integration of social cues and individual experiences during instrumental avoidance learning

**DOI:** 10.1371/journal.pcbi.1008163

**Published:** 2020-09-08

**Authors:** Philip Pärnamets, Andreas Olsson

**Affiliations:** 1 Division of Psychology, Department of Clinical Neuroscience, Karolinska Institutet, Stockholm, Sweden; 2 Department of Psychology, New York University, New York, New York, United States of America; Oxford University, UNITED KINGDOM

## Abstract

Learning to avoid harmful consequences can be a costly trial-and-error process. In such situations, social information can be leveraged to improve individual learning outcomes. Here, we investigated how participants used their own experiences and others’ social cues to avoid harm. Participants made repeated choices between harmful and safe options, each with different probabilities of generating shocks, while also seeing the image of a social partner. Some partners made predictive gaze cues towards the harmful choice option while others cued an option at random, and did so using neutral or fearful facial expressions. We tested how learned social information about partner reliability transferred across contexts by letting participants encounter the same partner in multiple trial blocks while facing novel choice options. Participants’ decisions were best explained by a reinforcement learning model that independently learned the probabilities of options being safe and of partners being reliable and combined these combined these estimates to generate choices. Advice from partners making a fearful facial expression influenced participants’ decisions more than advice from partners with neutral expressions. Our results showed that participants made better decisions when facing predictive partners and that they cached and transferred partner reliability estimates into new blocks. Using simulations we show that participants’ transfer of social information into novel contexts is better adapted to variable social environments where social partners may change their cuing strategy or become untrustworthy. Finally, we found no relation between autism questionnaire scores and performance in our task, but do find autism trait related differences in learning rate parameters.

## Author summary

People learn about dangers in their environment by directly interacting with it or indirectly from social sources. While what is dangerous in an environment can change, many times one’s social context is relatively stable. Surprisingly little work has investigated how people learn to avoid dangers by combining their own experiences with social advice in repeated settings. Combining behavioral testing and computational modeling, here we show that people blend social cues with information from their own experiences. When entering a new environment, people utilize stored information about how good advice a social partner gives to improve their choices. Crucially, however, we show that people don’t entirely rely on social advice even when that is the only valid information they have to base their choice on. Using simulations we show that while this tendency to not fully rely on social advice is less than optimal in our experimental task, it might be adaptive in settings where social partners become unreliable. Increasing our understanding of how people learn to avoid dangers using individual experiences and social information furthers the development of ecologically valid models of value-based learning and decision-making.

## Introduction

Learning about threats and dangers in the environment is important for survival across species. Many species, including humans, engage in social learning to leverage the knowledge other conspecifics may have acquired about the environment [1–4]. This allows individuals to avoid potentially costly trial-and-error learning and is under many conditions adaptive [2, 5] and scaffolds general cognitive capacities for associative learning [1]. Trial-and-error learning is not only costly in terms of time or energy expended searching for rewards, but can be especially so in a potentially dangerous environment where selecting the wrong action or holding false beliefs may be detrimental to an individual’s survival. Social learning sometimes takes the form of acquiring the behavior of a demonstrator, for example through imitation or teaching [6–9]. Another way of learning about a conspecific’s past experiences is through paying attention to their gestures or other bodily expressions that might signal risks and rewards in the environment [10–13]. In humans, the face is a particularly potent source of such signals, capable of carrying cues with varied informational content [14–16]. Little is known, however, about how well humans can leverage such signals to improve their decision making to learn to avoid harmful consequences and what the computational mechanisms underlying those abilities are. To investigate this, we used an instrumental aversive learning task where participants, prior to making decisions between two potentially harmful options, observed the gaze cues and fearful facial expressions made by different social partners.

To date a small body of work has investigated how social advice in the form of gaze cues and individual experiences are integrated during instrumental decision making [10, 13]. Social information in instrumental learning tasks is instead commonly represented by indicating the choice of a confederate demonstrator, sometimes coupled with an image of the demonstrator [8, 9] and sometimes without [17, 18]. If the outcome of the demonstrator’s choice is not represented, the choice can be understood as presenting advice similar to how a gaze cue can be interpreted as signaling about the cued option. In an early groundbreaking study researchers investigated the effects of volatility in social advice [17]. Participants made choices between two probabilistically rewarding options. Prior to each choice one option was highlighted representing the choice of a confederate. The confederate was performing another task to that of the participants which entailed that sometimes their choice would be helpful and sometimes unhelpful as advice to participants. Results showed that social and reward information were tracked in separate neural substrates and then integrated during decision-making. Studies investigating learning from gaze cues have also manipulated volatility [10, 13]. In these studies the reliability of a single social partner’s gaze cues was manipulated while participants made choices between different probabilistic alternatives. The key finding from these two studies was that, especially under conditions of high volatility, participants with high levels of autistic traits exhibited differences in how social information was integrated compared to participants with lower levels and, consequently, performed worse on the task.

Humans are very skilled at ascertaining the target of others’ gaze [19], owing in part to the physiological makeup of the human eye with a dark pupil and light sclera [20]. Indeed, previous work has shown that people automatically orient to gaze from an early age [21], and that in adults, this orienting has effects on both attention [22, 23] and preferences [24, 25]. In a standard gaze-cuing paradigm, a face is first presented facing and gazeing forward followed by a gaze shift. A target then appears beside the face. If the target appears at the cued location, recognition is significantly faster indicating covert attentional shifts facilitated by the social cue. Such immediate attentional effects have also been demonstrated using counter-predictive gaze cues [23, 26]. While there is ample evidence for the immediate attentional and evaluative effects of gaze cues, their use as inputs to evaluate decisions between options remain understudied, particularly in harmful contexts.

Previous work on learning from social advice and gaze cues has investigated the social learning problem of estimating how reliable a single individual is in a fixed decision context [10, 13, 17]. However, it does not address another feature of natural environments, namely that people typically encounter social partners across multiple decision contexts. For example, if a person is good at advising which stalls have safe food during the *Feast of San Gennaro* in Little Italy, they might also be good at advising which dumpling spots in nearby Chinatown are safe to visit. This suggests that remembering how likely it is that a social partner gives accurate advice, or is able to signal safe options, is necessary to maximally leverage their knowledge during repeated encounters. However, a study where participants could learn from from a demonstrators’ choices found mixed results regarding if participants transfer information about demonstrators to novel contexts [8]. In the current study, we allowed participants to learn from multiple partners differing in their ability to reliably cue alternatives. Additionally, partners were re-encountered multiple times during the experiment in contexts where participants faced novel choice options. This design allowed us to capture several crucial features of the human social environment, one where we typically must learn to track the utility of multiple individual partners and transfer that information to decisions about actions in novel contexts.

Considerable research has investigated how the combination of emotional expressions and gaze direction can confer values to objects, so-called social referencing [11, 12, 27]. This work has shown that value can be transferred to objects being referenced [11]. However, how gaze cues and emotional expressions interact to shape instrumental decisions has not been investigated. Avoidance-oriented emotional expressions, such as fear, are more quickly identified when gaze is also averted indicating that gaze direction and emotional expressions are contextually processed [28]. Similarly, the attentional effects of gaze cues are amplified with fearful expressions as these represent potentially more significant signals to the perceiver [12, 29]. Given these findings, we allowed some of the partners in the present study to express fear while giving gaze cues. We reasoned that this is a naturalistic signal for danger [30, 31]. If participants pay extra attention to gaze cues from partners with fearful expressions, then this should bias their decisions.

**Fig 1:**
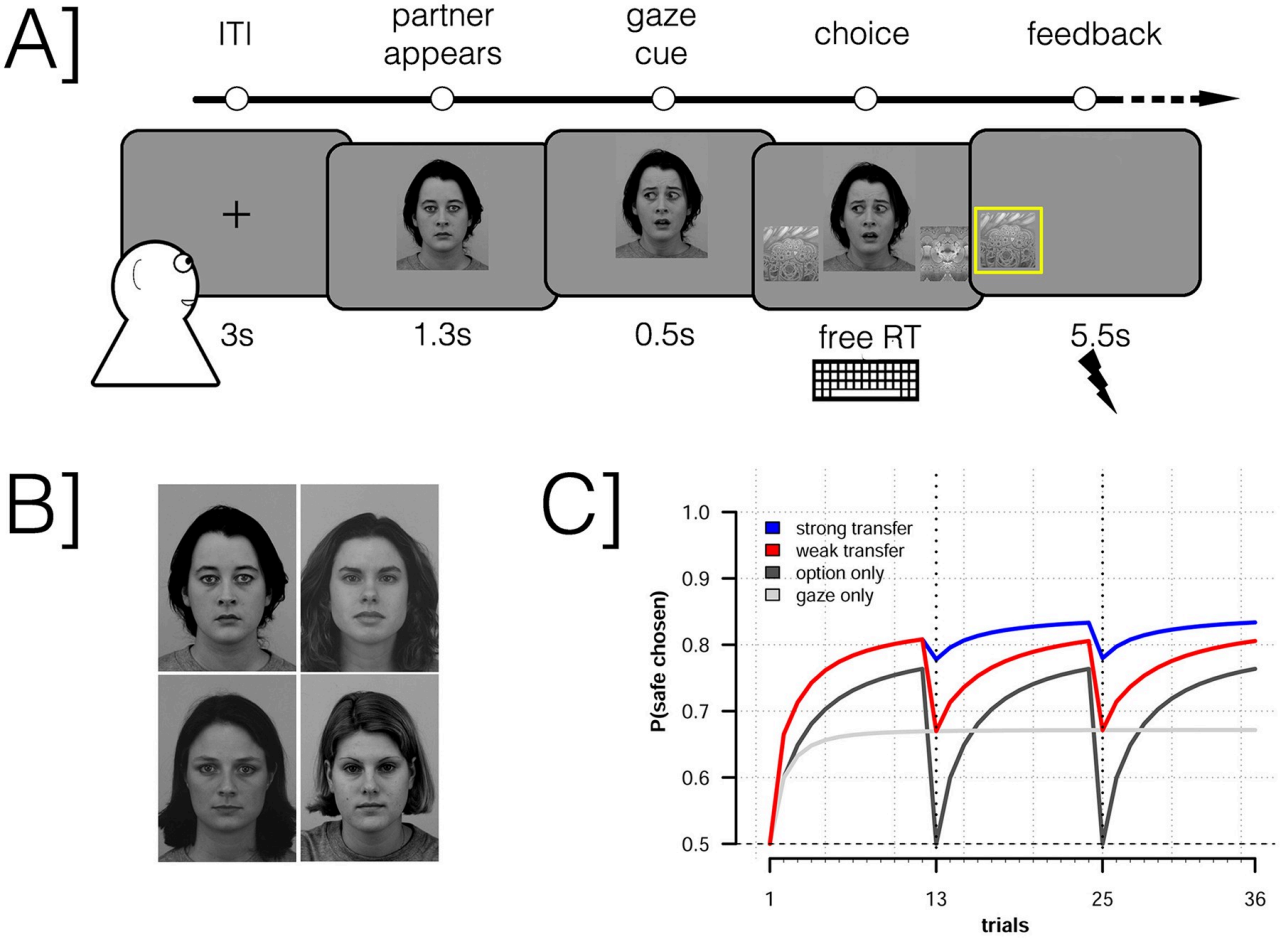
Methods overview. A: Example trial structure. Participants view a fixation cross, followed by the appearance of the partner of the current block of 12 trials. The partner makes a gaze cue and shortly thereafter the two alternative options appear, randomly assigned to the left or right side of the screen. Once participants have made their choice, the selected alternative remains onscreen, highlighted for 5.5s. At the end of this period the participants may receive a shock. Displaying KDEF images AF14NES, AF14AFS. B: The four social partners used in the experiment, each was randomly assigned to one condition for each participant. Displaying KDEF images AF01NES, AF14NES, AF26NES, AF29NES. C: *A priori* predictions of models **1-4** for the case when partners are predictive. Models **2-4** cache the learned value of the social partner when transferring between blocks that feature novel choice options. This allows them to predict above chance performance on the first trial by partially relying on the social partners’ gaze cue.

We present results from an experiment investigating instrumental learning under threat of shock while viewing different partners giving either predictive or random gaze cues coupled with either neutral or fearful facial expressions (see Fig 1). Participants were placed in one of two conditions; in the naïve condition participants were not told anything about the role of the social partners and simply told that at the start of each trial they would see a face on the screen. In the instructed condition we informed participants that the faces represented social partners some of who would have information about what choice options were dangerous, although no specific information was given. Participants were not otherwise told anything if the social partners were human or computer controlled. Our design allowed us to explore if prior knowledge about the potential relevance of the social partners impacted behavior in our task, although we did not formulate any directional hypotheses concerning the effects of instruction. Participants were also assessed on their autistic traits by filling out an Autism Questionairre (AQ) at the end of the experiment [32]. We expected to find task related deficiencies consistent with previous findings in the literature related to participants’ AQ scores. We also explored if participants’ AQ scores related to parameters derived from a reinforcement learning model describing their learning and decision making in our task.

We found that participants’ behavior was best explained by a reinforcement learning model which assumed that they learned the probabilities of the options being safe as well as of the partners accurately signaling safety. Participants then combined the resulting value estimates to make decisions, while also following advice from partners making a fearful expression to a greater degree through a fixed bonus to their decision making. Estimates of partners’ probability of giving accurate cues was transferred into novel contexts. We additionally explored the ecological validity of participants’ transfer of information between contexts, attempting to explain apparent suboptimalities in participants’ learning strategy by simulating model outcomes in a variety of environments.

## Results

### Choice behavior

**Fig 2:**
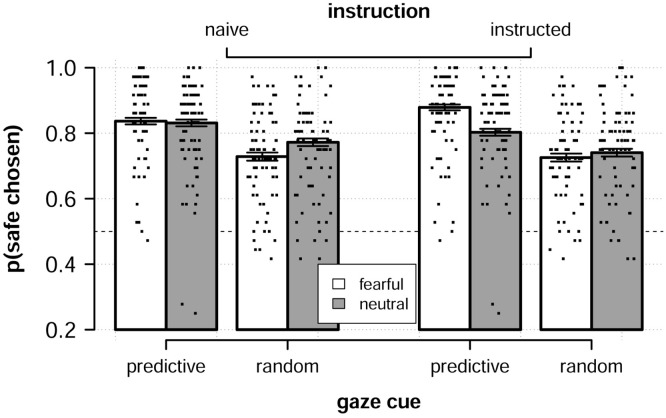
Choice behavior. Average proportion of safe choices across all participants split by conditions and instruction. Error bars are standard errors. Individual dots represent participant averages.

We first analyzed choice behavior across all participants using a logistic generalized mixed model with partner reliability (*predictive* or *random*), emotional expression (*fearful* or *neutral*) and instruction condition (*naïve* or *instructed*) as predictors together with their interactions. We defined a choice as being safe if the option with the lowest shock probability was chosen, see Fig 2 for descriptive results. We found that participants made more safe choices when facing predictive compared to random partners (*b*_*reliability*_ = 0.67, *SE* = 0.10, 95% CrI = [0.48, 0.85], *pd* ≈ 1.0) and this effect did not robustly interact with instruction (*b*_rel*instr_ = 0.11, *SE* = 0.18, 95% CrI = [-0.25, 0.45], *pd* = .73). Indeed, we found no average difference in proportion safe choices depending on instruction (*b*_*instruction*_ = 0.0, *SE* = 0.14, 95% CrI = [-0.28, 0.28], *pd* = 0.5). Together this shows that participants readily learned to use the predictive partners’ gaze cues to improve their decision-making but that receiving instructions about the partners had no effect on this ability.

We found mixed results regarding the effects of partners giving fearful or neutral emotional expressions. There was no general increase in safe choices based on partners’ emotional expressions (*b*_*emotion*_ = 0.08, *SE* = 0.071, 95% CrI = [-0.06, 0.22], *pd* = 0.87), but instead we found an interaction between partner reliability and emotional expression (*b*_rel*emot_ = 0.51, *SE* = 0.14, 95% CrI = [0.23, 0.78], *pd* ≈ 1.0). The interaction showed that participants tended to make more safe choices to fearful compared to neutral partners in the predictive condition, while making fewer safe choices to fearful compared to neutral partners in the neutral condition. It is also probable that emotional expression increased safe choices more for participants given additional instructions (*b*_emot*instr_ = 0.26, *SE* = 0.14, 95% CrI = [-0.017, 0.53], *pd* = 0.97), but we note that the credible intervals overlap with zero rendering strong conclusions inappropriate. Further, in light of the lack of effects on average performance, this increase of safe choices to fearful partners appears to have been “compensated” by a decrease in safe choices to neutral partners additionally complicating interpretation of this effect. In the remainder of the paper, unless otherwise specified, we analyze data collapsed by participant instruction.

A key prediction was that if participants would retain and use previously learned information about the social partner, they should be above chance choosing the safe option on the first trial when facing reliable partners a second and third time. Therefore, we examined participants’ choices on the first trial of the second and third blocks to each partner. We found that participants indeed were above chance on these trials when facing predictive partners. In the second block, participants chose the safe option above chance when facing the predictive fearful partner (*M* = 0.79, logistic regression *b* = 1.25, *SE* = 0.26, 95% CrI = [0.76, 1.78], *pd* ≈ 1.0) and when facing the predictive neutral partner (*M* = 0.68, *b* = 0.72, *SE* = 0.24, 95% CrI = [0.26, 1.20], *pd* = 0.999). In the third block, the same results were observed, with participants choosing the safe option above chance both when facing the predictive fearful partner (*M* = 0.84, *b* = 1.54, *SE* = 0.28, 95% CrI = [1.0, 2.12], *pd* ≈ 1.0) and when facing the predictive neutral partner (*M* = 0.70, *b* = 0.83, *SE* = 0.24, 95% CrI = [0.37, 1.30, *pd* ≈ 1.0). In contrast, this was not observed for the two random partners in block two (fearful, *M* = 0.47, *b* = -0.11, *SE* = 0.22, 95% CrI = [-0.54, 0.33], *pd* = 0.695; neutral, *M* = 0.52, *b* = 0.072, *SE* = 0.22, 95% CrI = [-0.35, 0.53], *pd* = 0.622) or in block three (fearful, *M* = 0.54, *b* = 0.17, *SE* = 0.22, 95% CrI = [-0.26, 0.61], *pd* = 0.778; neutral, *M* = 0.54, *b* = 0.17, *SE* = 0.22, 95% CrI = [-0.26 0.62], *pd* = 0.776).

In sum, participants learned that some social partners were reliably predictive and used that information to make more safe choices. Furthermore, they retained their learning about the social partners and were able to benefit it when proceeding through the experiment and encountering novel choice options. The next question was how they achieved this.

### Computational modeling

To understand participants’ learning and decision-making in the experiment, we analyzed their trial-by-trial decisions with the help of reinforcement learning (RL) models (see Methods and Table 1). All models, except for the the null models **1-2**, assumed that participants learned both the probability of each option being safe and the reliability of the social partners (the probability of each social partner giving good advice). In the null models, by contrast, participants only learned from one source of information. We formulated a *weak transfer* and *strong transfer* version of the different models. Transfer refers to how the learning about partners’ reliability affects choices between new options between blocks (see Fig 1C). Under weak transfer, participants are assumed to cache the reliability of each social partner between blocks. By contrast, under strong transfer, participants additionally allow the reliability of the social partner influence their initial estimates of novel options.

**Table 1:** Overview of models compared and their respective free parameters. ELPD = expected log predictive density (larger is better). Winning **model 9** highlighted in bold.

Model	Parameters	Transfer	#	ELPD
Option only	*β*, *α*_+/−,*opt*_	-	1	-4634
Gaze only	*β*, *α*_+/−,*partner*_	Weak	2	-6219
Equal weighting	*β*, *α*_+/−,*opt*_, *α*_+/−,*partner*_	Weak	3	-4239
		Strong	4	-4340
Variable weighting	*β*, *α*_+/−,*opt*_, *α*_+/−,*partner*_, *ω*	Weak	5	-4239
		Strong	6	-4318
Emotion weighting	*β*, *α*_+/−,*opt*_, *α*_+/−,*partner*_, *ω*_*n*_, *ω*_*f*_	Weak	7	-4164
		Strong	8	-4329
Emotion bonus	*β*, *α*_+/−,*opt*_, *α*_+/−,*partner*_, *ω*, *θ*	**Weak**	**9**	**-4145**
		Strong	10	-4277
Arbitration	*β*, *α*_+/−,*opt*_,*α*_+/−,*partner*_, *γ*	Weak	11	-4188
		Strong	12	-4306
Arbitration emotion bonus	*β*, *α*_+/−,*opt*_,*α*_+/−,*partner*_, *γ* *θ*	Weak	13	-4155
		Strong	14	-4239

The models also differed in how option and partner information was combined and if the partners’ emotional expression was taken into account or not. We assumed that participants weighted option values according to a weighting parameter, *ω*, and combined this with the value of the partners’ advice (1 − *ω*). The weighting parameter was fixed in models **3-4** and variable in in models **5-14**. Several of the models additionally modeled how participants’ decisions might have changed in response to the partners’ emotional expression. In models **7-8**, the weighting parameter differed depending on the partners emotional expression. In models **9-10** and **13-14** emotional expression was taking into account through a bonus parameter, *θ*, conferring a fixed value bonus to the advised option from a partner making a fearful facial expression.

**Fig 3:**
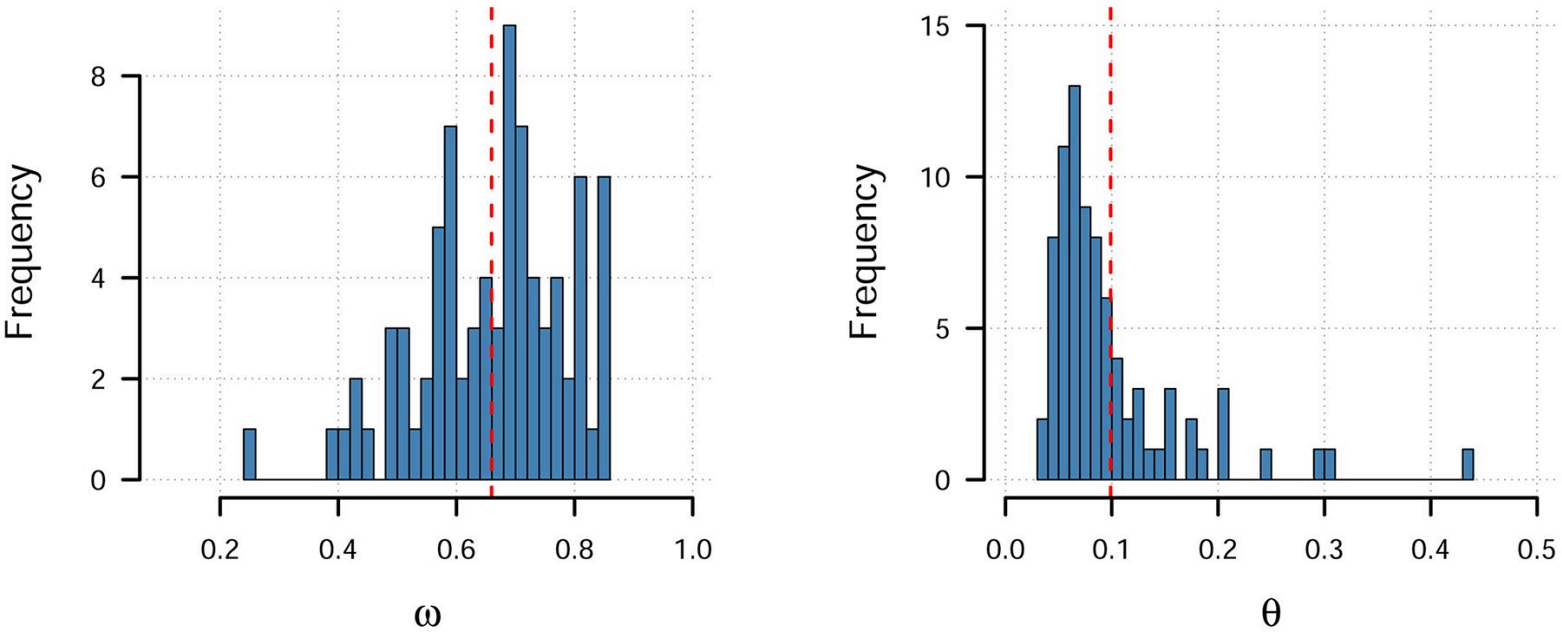
Parameter histograms. Histograms of the per participant average posterior parameter estimates of the weighting parameter *ω* and the emotion bonus parameter *θ* from model **9**. Dashed red line corresponds to the estimated population average parameter value.

To compare the models we used leave-one-out cross-validation (LOO-CV) to compute the expected log predictive density (ELPD) of each model which quantifies the predictive accuracy of the models [33], analogous to what information criteria estimate. The results of the model comparison are summarized in Table 2. Model **9** emerged as the winning model. This model used weak transfer and it further assumed that each participant varied in their relative weighting of option and partner information and that fearful expressions conferred a fixed bonus to the option advised (see Methods, Eq (7)). The distribution of the average per-participant estimates of the weighting parameter *ω* ranged between 0.26 and 0.85, with a clear majority of participants (72 of 81) being fit with a greater weighting for options over partner advice (*ω* > 0.5), see also Fig 3. The emotion bonus parameter was positive for all participants, ranging from 0.038 to 0.44 (see Fig 3), meaning that all participants were on average estimated to be more likely to follow the partners’ advice if they made a fearful expression. Estimated population parameter values for all parameters are presented in Table 3. In the S1 Text we report additional exploratory analyses of model parameters depending on instruction condition.

**Table 2:** Full model comparison. Δ ELPD is the difference in expected log predictive density (ELPD) between each model and the winning model. SE Δ approximates the standard error of the difference between each model and the winning model. ELPD and SE ELPD give each model’s expected log predictive density and their standard error. *p*_*eff*_ and SE *p*_*eff*_ estimates the effective number of parameters in each model and the standard error of this estimate.

Model #	Δ ELPD	SE Δ	ELPD	SE ELPD	*p*_*eff*_	SE *p*_*eff*_
9	0.00	0.00	-4144.84	167.26	275.81	8.20
13	-10.30	13.28	-4155.14	165.89	282.51	8.32
7	-19.17	9.76	-4164.01	164.33	291.55	8.70
5	-27.56	10.95	-4172.40	165.51	248.78	7.03
11	-42.75	16.90	-4187.59	164.61	246.28	7.53
3	-93.88	19.93	-4238.72	160.93	245.42	8.31
14	-120.99	25.59	-4265.83	164.07	326.08	10.61
10	-132.25	23.64	-4277.10	163.92	319.53	10.02
12	-160.74	30.47	-4305.58	162.24	298.84	10.10
6	-173.65	30.78	-4318.49	160.96	297.55	9.47
8	-184.41	31.01	-4329.26	161.88	330.51	9.66
4	-195.42	31.52	-4340.26	165.06	270.24	8.17
1	-488.73	53.87	-4633.57	178.26	155.05	5.32
2	-2074.57	138.57	-6219.41	92.31	118.03	7.24

**Table 3:** Population-level average parameter estimates and 95% credible intervals from the posterior of the winning model 9.

Parameter	Mean	2.5%	97.5%
*β*	0.25	0.23	0.28
*ω*	0.68	0.62	0.73
*α*_+,*opt*_	0.53	0.43	0.63
*α*_−,*opt*_	0.19	0.13	0.27
*α*_+,*partner*_	0.45	0.35	0.56
*α*_−,*partner*_	0.33	0.26	0.41
*θ*	0.069	0.039	0.11

Model **13** came close second in the comparison, indeed, within one standard error of difference to the wining model **9** but with a higher amount of effective parameters. This model implemented a dynamically changing weighting between option and partner information based on past prediction errors (see Methods). In addition to this dynamic arbitration a fixed bias parameter, *γ* was also applied. The per participant fitted values of *γ* were found to strongly correlate with the per participant weighting parameter *ω* from model **9** (robust correlation, *r* = -0.91, *SE* = 0.021, 95% CrI = [-0.94, -0.86], *pd* ≈ 1.0), suggesting close similarities in the roles that those parameters were playing in their respective models. While we discuss and interpret model **9** in the remainder of the results, we return to the question of adjudicating between these two models in the discussion.

**Fig 4:**
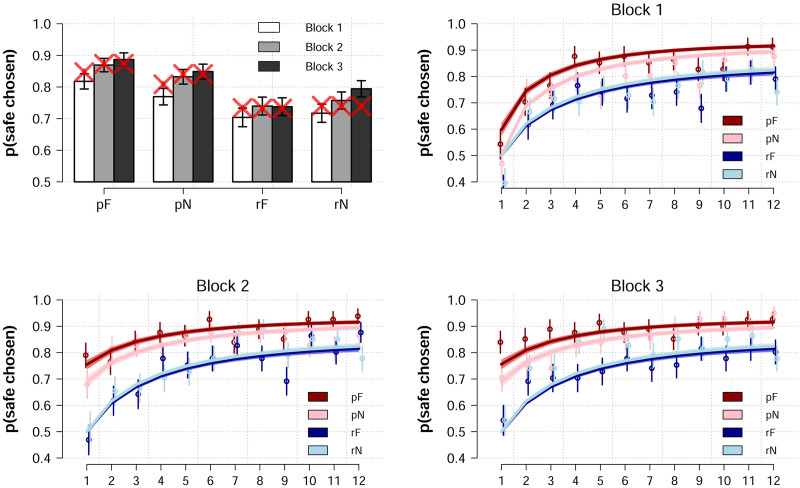
Posterior predictions. Average proportion of safe choices across all participants, split by block and condition (p = predictive, r = random, F = fearful, N = neutral). Error bars are standard errors. Model predictions using full posterior of each participants’ parameter estimates each simulated to 500 times to new data matching the parameters of our experiment. Shaded regions represent 95% predictive intervals.

Finally, we simulated decision trajectories from each participants’ best fitting parameters from model **9** to new data, simulating each participant 500 times. These posterior model predictions are plotted by-block and trial-by-trial in Fig 4, and illustrate how the model captures and generalizes the empirical data patterns including between block transfer of social information and both average and trial-by-trial decision probabilities.

#### Between block transfer of partner information

Model comparisons bore out that models using weak transfer better described participants data than models using strong transfer. In other words, participants cached partner reliability when transitioning into new decision context, but did not let that influence their initial estimate of options’ safety. However, *a priori* simulations (see Fig 1C) clearly indicated that strong transfer represents a better algorithm for participants in this experiment (i.e. it makes more safe choices).

To explore the conditions under which weak transfer and strong transfer is better with respect to partners’ reliability, we conducted simulations. We simulated each type of transfer 1000 times for all combinations of twelve evenly spaced samples of all learning rates *α*_+,*partner*_, *α*_−,*partner*_, *α*_+,*option*_, *α*_−,*option*_ in [0.1, 0.8] and of the temperature parameter *β* in [0.2, 1], using a fixed *ω* of 0.5 (i.e. the equal weighing models **3** and **4**).

**Fig 5:**
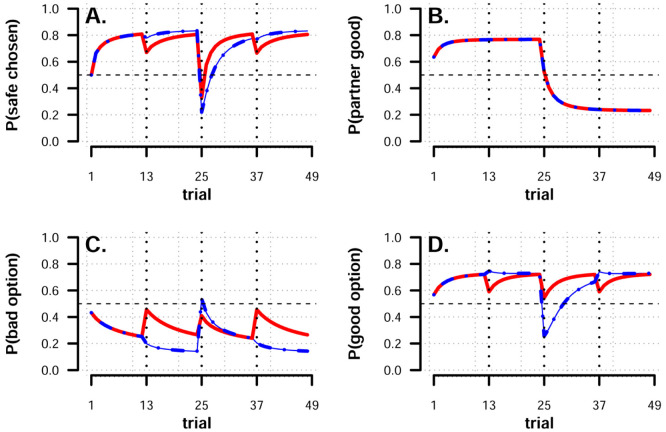
Simulation results, partner reliability reversal. A: Average difference in proportion safe choices between weak (red, solid) and strong (blue, dashed) transfer models when social partners reverse advice (on trial 25). B: Each model’s estimate of the partner. C-D: Each model’s estimate of the safety of the bad and good option respectively. For panels B-D, estimates are plotted following learning on the trial depicted. Dotted vertical lines indicate new decision environments (blocks) where novel options are presented. The horizontal dashed line represents chance performance.

We simulated four blocks of 12 trials, where the partner always cues the bad option during the first two blocks and then cues the good option in the last two blocks (Fig 5). As in our experiment, options are assumed to be novel in each block. This means that the first two blocks simply simulate our experiment with a *predictive* partner. The transition between the second and the third block in the simulation represents a situation where a previously trusted source of information completely reverses its signals. This can arise, for example, if a partner loses track of the environment but fails to realize it or if they continue to track the environment but change how they communicate their information about it.

We examined the proportions of safe choices made during the second and third blocks by calculating the differences and ratios between models. During the second block, replicating the observations from Fig 1C, the difference between the models was on average 5.2 percentage points (p.p.) (*Q*_10_ = −0.35p.p., *Q*_90_ = 10.2p.p.), confirming that the strong transfer model is generally better when the partner’s ability to give good advice is stable. This translates to a ratio of safe choices between the models of 1.06 (*Q*_10_ = 0.995, *Q*_90_ = 1.13). During the third block, by contrast, the relationship between the models was reversed, such that the difference strongly favored the weak transfer model (*M* = 10.1p.p., *Q*_10_ = 0.46p.p., *Q*_90_ = 22.6p.p.). The difference between the models in the third block was even starker when considering the ratios: 1.60 (*Q*_10_ = 1.0, *Q*_90_ = 2.0). As is evident from Fig 5a, differences between the weak and strong transfer models diminished as agents learned about the environment. To illustrate this, we restricted our comparison only to the first half of the third block. In this case the difference between the models increases to (*M* = 14.7p.p., *Q*_10_ = 2.0p.p., *Q*_90_ = 29.9pp), with an average ratio 2.08 (*Q*_10_ = 1.03, *Q*_90_ = 2.75). In the fourth block, as can be seen in Fig 5A, the strong transfer model once again performs better owing to the stability in the partners’ signal. Finally, as seen in Fig 5B, 5C and 5D, the differences between the models’ internal estimates lie not with what is estimated about the partner, but what is learned about the options. This suggests that differences between the models should be exacerbated especially for agents with low learning rates about options, in particular for negative prediction errors *α*_−,*opt*_. The reason for this is that because following a reversal of the way the partner communicates, under strong transfer the “bad” option will now have (wrongly) been assigned a high probability of being good. This means that it will be chosen more often. When choosing the bad option the agent will experience negative prediction errors and if the learning rate from these is low, then it will take longer time for the new information to override the old. In Fig A in S1 Text we plot the differences in proportion safe choices between the models for each simulated parameter combination, and visually examining the resulting plots bears out this prediction.

**Fig 6:**
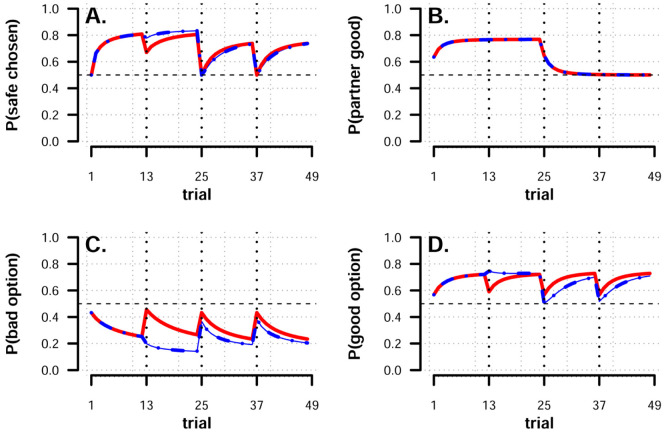
Simulation results, partner becomes random. A: Average difference in proportion safe choices between weak (red, solid) and strong (blue, dashed) transfer models when social partners stop giving informative advice (on trial 25). B: Each model’s estimate of the partner. C-D: Each model’s estimate of the safety of the bad and good option respectively. For panels B-D, estimates are plotted following learning on the trial depicted. Dotted vertical lines indicate new decision environments (blocks) where novel options are presented. The horizontal dashed line represents chance performance.

We also simulated a second situation where partners instead of reversing their advice in the third block instead begin guessing at random (Fig 6). In this situation differences between the models are much smaller, but even in this case the weak transfer model makes slightly safer choices on average (*M* = 1.6p.p., *Q*_10_ = −2.0p.p., *Q*_90_ = 5.6pp), with an average ratio 1.03 (*Q*_10_ = 0.97, *Q*_90_ = 1.09). Like previously, restricting the comparison to the first half of the block increases the differences between the models (*M* = 2.2p.p., *Q*_10_ = −1.6p.p., *Q*_90_ = 6.7pp), with an average ratio 1.04 (*Q*_10_ = 0.97, *Q*_90_ = 1.11). In this simulation, the strong transfer model has no advantage over the weak transfer model going into the fourth block and a slight advantage remains for the weak transfer model, owing primarily to differences in option estimates (see Fig 6B, 6C and 6D).

### Choice response times

**Fig 7:**
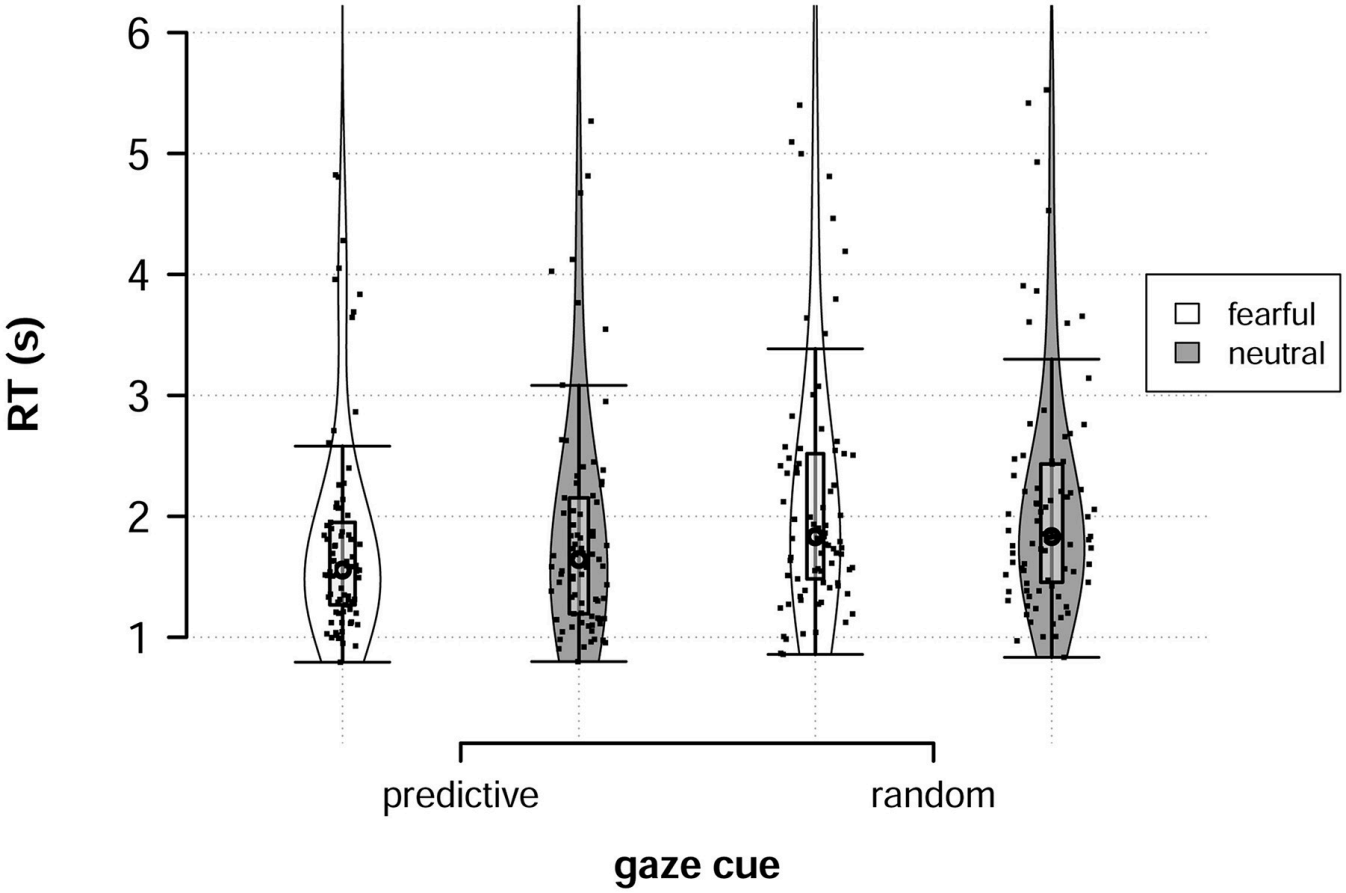
Choice response times. Response times to the four partners. Boxplots show medians (circle) and 25th and 75th percentiles, while hinges represent the interquartile range. Points represent participant average response times.

We analyzed participants choice response times throughout the experiment, as response times provide additional information about cognitive processing not contained in decisions alone. Participants’ response times when facing the Predictive Fearful partner were *M* = 1.9s, *SD* = 2.4; when facing the Predictive Neutral partner *M* = 2.0s, *SD* = 2.8; when facing the Random Fearful partner *M* = 2.4s, *SD* = 3.8, and, when facing the Random Neutral partner *M* = 2.2s, *SD* = 2.6, see also Fig 7.

We fit a regression model using an ex-Gaussian likelihood to account for the skewed nature of response times [34], with partner partner reliability and emotional expression as predictors on both the *μ* and *τ* components of the distribution. In the ex-Gaussian, *μ* reflects shifts in the mean of the distribution and *τ* reflects both shifts in the mean and variance. We found that partner reliability negatively affected both *μ* (*b*_*μ*,*reliability*_ = -0.26, *SE* = 0.029, 95% CrI = [-0.32, -0.21], *pd* ≈ 1.0) and *τ* (*b*_*τ*,*reliability*_ = -0.19, *SE* = 0.022, 95% CrI = [-0.24, -0.15], *pd* ≈ 1.0). Participants were faster when responding to predictive partners and showed less variance in their response times. Additionally, we found an interaction between partner reliability and emotional expression on the *μ* parameter (*b*_*μ*,rel*emot_ = -0.15, *SE* = 0.067, 95% CrI = [-0.29, -0.03], *pd* = .99), capturing that responses were faster to fearful partners when they were predictive but slower when partners were random.

### AQ scores

We investigated if participants’ autism questionnaire scores (AQ) correlated with their performance to either the partners who reliably gave predictive gaze cues or to the ones that didn’t. Average AQ score was *M* = 19.7, *SD* = 6.4, range = [8, 39]. We found no correlation between AQ sores and average number of safe choices when facing predictive partners (robust correlation, *r* = 0.06, *SE* = 0.11, 95% CrI = [-0.16, 0.28], *pd* = 0.702). Similarly, we found no correlation when facing random partners (robust correlation, *r* = 0.05, *SE* = 0.11, 95% CrI = [-0.17, 0.27], *pd* = 0.651). In sum, we found no relationship between AQ scores and performance in our task.

**Fig 8:**
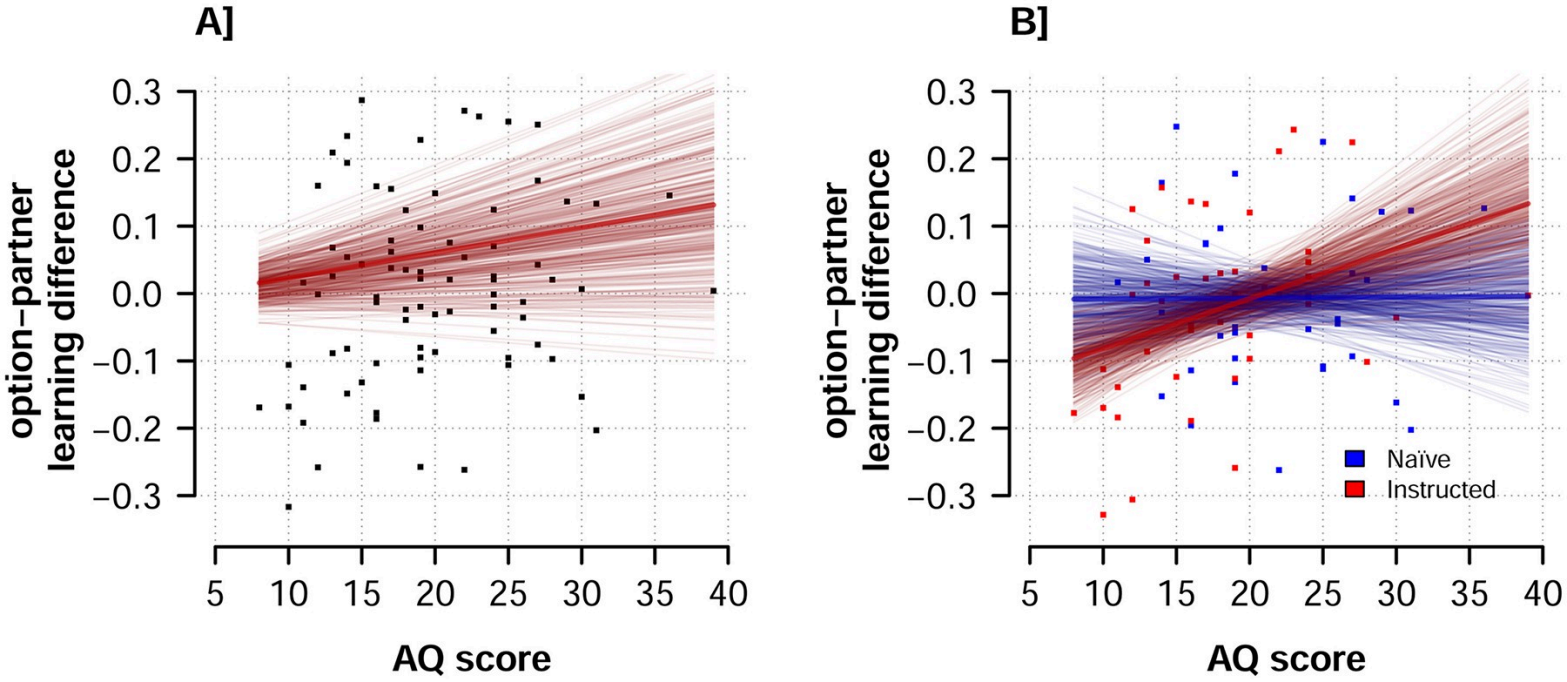
AQ scores and learning rates. A: Correlation between option and partner learning rate difference and participant AQ-scores. B: Interaction between AQ scores and instruction condition on option-partner learning rate difference. Bold line depict the regression line derived from the mean posterior parameter estimates, and thin lines show regression line derived from 400 draws from the posterior distribution of parameter estimates, indicating uncertainty around the mean estimate.

However, it is possible that participants acquire information about options and partners differently in a way that correlates with the AQ scores [13]. To capture this we constructed a measure by subtracting the average partner learning rates (i.e. average of *α*_+,*partner*_ and *α*_−,*partner*_) from the analogous average of the option learning rates for each participant using the posterior mean. We correlated this option-partner learning rate difference with participants’ AQ scores. We found a small positive correlation, such that participants with higher AQ scores also exhibited larger differences between option and partner learning rates (robust correlation, *r* = 0.19, *SE* = 0.11, 95% CrI = [-0.04, 0.39], *pd* = 0.951), see also Fig 8A. As this finding was exploratory, we assessed its robustness by computing similar correlations for other models and found consistent results (see S1 Text).

We further explored the relationship of AQ scores to the option-partner learning rate difference by considering the possible role of instruction condition. It is possible that the top-down manipulation of instruction could affect the relative weights placed on learning from options versus social partners. To test this possibility we regressed the option-partner learning rate difference on standardized AQ scores and a variable indicating instruction condition. We found a probable main effect of AQ scores (*b* = 0.024, *SE* = 0.015, 95% CrI = [-0.005, 0.053], *pd* = 0.949) and no main effect of instruction condition (*b* = -0.003, *SE* = 0.029, 95% CrI = [-0.058, 0.054], *pd* = 0.54). However, these effects were qualified by an interaction effect (*b* = 0.046, *SE* = 0.029, 95% CrI = [-0.011, 0.103], *pd* = 0.945), which we plot in Fig 8B. Together, these findings indicated that the relationship between option-partner learning rate difference likely emerged only in the instructed condition and not in the naïve condition.

### Partner helpfulness

**Fig 9:**
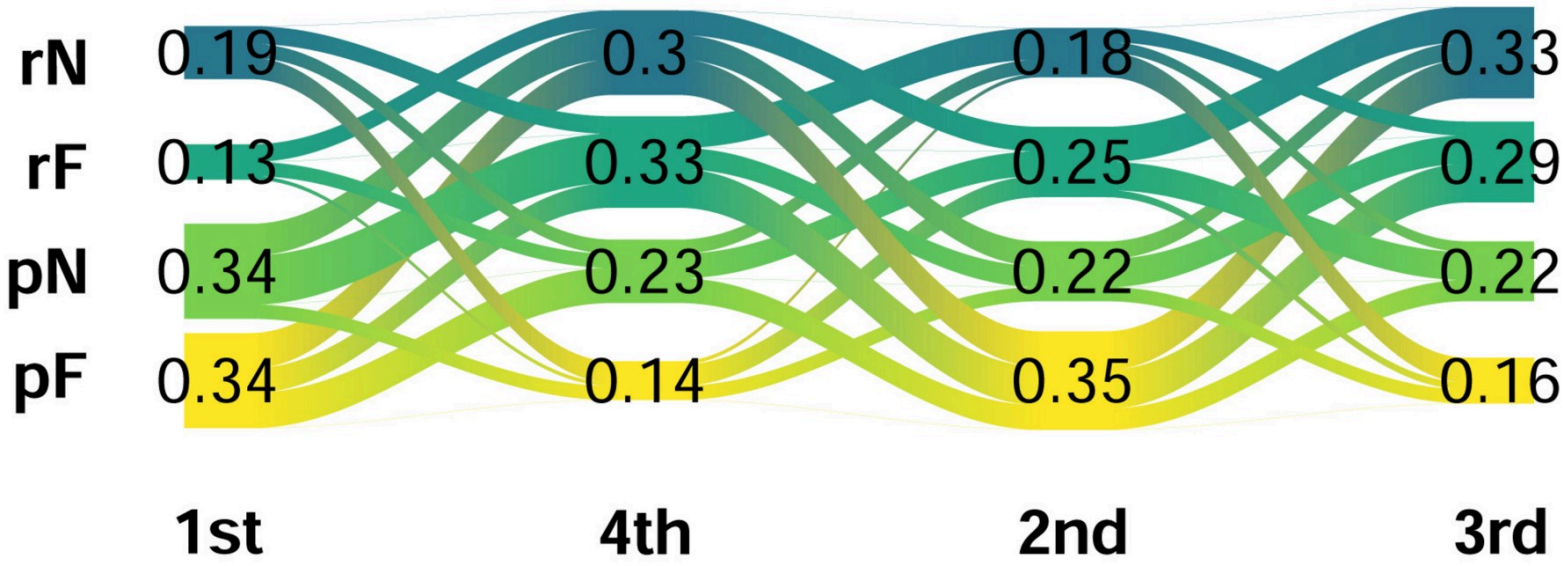
Ranking of partners at end of experiment. Proportion of participants selecting each partner at each stage of the forced choice helpfulness task. rN = random Neutral, rF = random Fearful, pN = predictive Neutral, pF = predictive Fearful.

Lastly we examined participants’ selections at the end of the experiment, where they made a series of forced choices to, in order, select which partner they thought was the most helpful (of 4), the least helpful (of 3 remaining) and finally most helpful from the final pair. This produced a rank ordering of partners for all participants. The results of participants selections can be seen in Fig 9. Overall, participants were fairly accurate at classifying one of the predictive partners as being most helpful (68%) and at classifying one of the random partners as least helpful (63%).

We explored the extent to which participants’ experiences and model predictions explained the rankings of the different social partners. We regressed social partner rank on each participants’ model derived final estimates of each social partner’s reliability together with the number of shocks each participant experienced from their choices in the presence of each partner (z-scored) together with their interaction using a multi-level ordered probit regression. The analysis indicated that both estimated partner reliability (*b* = 0.68, *SE* = 0.27, 95% CrI = [0.15, 1.21], *pd* = 0.993) and number of shocks experience (*b* = -0.18, *SE* = 0.065, 95% CrI = [-0.31, 0.051], *pd* = 0.996) influenced rankings, but not their interaction (*b* = -0.084, *SE* = 0.30, 95% CrI = [-0.68, 0.50], *pd* = 0.611).

We next considered if the each participant’s *θ* estimate from model **9** was associated with ranking fearful over neutral partners. We constructed an index ranging between 0 and 2 indicating if the fearful partner has been ranked over the neutral for only the predictive or random partners, neither or both. We found a probable effect of *θ* indicating it that participants with higher tendency to follow fearful partners’ advice also ranked fearful partners as being more helpful (*b* = 0.26, *SE* = 0.15, 95% CrI = [-0.023, 0.55], *pd* = 0.965), but we note that the 95% credible intervals include zero cautioning against strong conclusions. In the S1 Text we report an additional analyses on rankings contrasting participants best fit by models **9** and **10**.

## Discussion

We investigated how human participants learn to avoid harmful choice options based on information from two sources: social (gaze) cues from partners with different reliability and trial-and-error learning. We found that participants readily acquired relevant predictive information from gaze cues and used this to improve their decision making. Participants learned to separately track and update multiple partners’ predictive value. Participants were, on average, as adept at learning from gaze cues spontaneously as when they had prior knowledge that partners would have predictive information, which is in line with research on human infants showing that gaze following emerges early in development [21], as well as work showing that gaze signaling frequently occurs in naturalistic context [16]. Using gaze cues as a source of social information is likely highly over-trained in adults, and suggests that participants were already highly prepared to attend to partners’ gaze cues as valid sources for social learning.

Participants’ learning and decision making was best explained by a reinforcement learning model which assumed that participants independently track the probability that options have good/bad outcomes and the reliability of the social partners’ advice. Participants weighted these sources differently to combine them when making decisions, but generally put greater weights on option information compared to partner information. The work presented here contributes to a growing literature investigating how social information is acquired and combined with experiential information [7–9, 13, 17, 18], and concords with a developing consensus that basic associative learning principles can be applied to understand a wide variety of adaptive social behaviors [35].

One question not settled in the present work arose from the close comparison between the winning model **9** and model **13**. This alternative model was an arbitration model which assumed that weights between options and partner advice were partially determined from the past prediction errors. In [18], where we derived model **13** from, arbitration was between information gained from two different types of observational learning—imitation and emulation. Imitation can be characterized as being more model-free while emulation as being more model-based. In our task participants had to weigh one source of social information against information derived from their own experience of the choice options, but both sources are essentially model-free. This difference may account for why the arbitration model did not best describe our participants data. Simulations similarly showed that the two models were not strongly distinguishable (see S1 Text). However, further experimentation, potentially targeting the neural correlates identified in [18] will be necessary to fully settle how participants weigh information in tasks like ours.

Our results showed that participants cache the probability of a partner giving good advice and use that information to guide decisions during subsequent encounters with that partner. This allows for a gradual improvement in the quality of participants’ decisions over time, even when facing choices between novel options. Indeed, participants decisions on the first trial when facing a predictive partner they had learned about were above chance. Despite caching partner estimates between blocks, participants did not fully capitalize on their previous learning when transitioning into a new context. Letting information from partners’ advice spill over to initial option estimates, as formalized in the strong transfer models, would have been a better strategy in our experiments. Why then didn’t participants adopt this strategy?

One reason for this apparent suboptimality in participants’ behavior might be that their strategy is tuned to the possibility of partners’ reliability varying when transitioning between choice environments. The way participants used cached social information, as formalized in the weak transfer formulation of our models, was shown to be a better strategy in conditions where partners could not be trusted to give reliable advice between contexts compared to that of the strong transfer models. We argue that participants’ behavior likely reflects expectations about social partners which they bring with them from their lives into the lab. If someone once suggested to you which of two Italian food stalls were safest to eat from, it might be a good idea to take their advice into consideration when choosing between two Chinese restaurants (as in the weak transfer models). However, you might be hesitant to completely take their word for it and assume that each restaurant is exactly as safe as they say it is (the strong transfer models). Indeed, both theoretical results and empirical findings on the cultural evolution of social learning have indicated the importance of switching between learning from experience and social learning [5, 36]. In volatile environments, reliance on social information can be maladaptive and lead to fitness loss compared to reliance on individual experience. In stable environments, the reverse is true. Our findings suggest that participants might not be switching between pure social and non-social learning strategies but rather using both to inform their decisions. Beyond understanding how participants solved this particular task, our findings highlight the importance of considering the ecological factors which shape human decision makers beyond the confines of the lab when interpreting the results from experimental tasks [37]. Additionally, loss or risk sensitivity [38], in addition to prior social experiences, might contribute to explaining why participants exhibit this particular transfer behavior by causing participants to overweight the prospect of aversive experiences. By not fully transferring social information, weak transfer models err on the side of caution. Nevertheless, further experimentation will be necessary to pinpoint if these are the responsible psychological mechanisms.

In addition to manipulating partner reliability, we also introduced an emotional expression manipulation. We expected that fearful facial expressions may improve decision-making, acting as ecologically valid signals of danger [12]. Our results supported this hypothesis. On average, participants made more safe choices when facing the predictive fearful partners compared to when face the predictive neutral ones. These tendencies appeared reversed for the random partners. We also observed choice response time patterns supporting the conclusions drawn from the choice data—participants responded fastest to the predictive fearful partners and slowest to the random fearful partner. The model that best described participants’ behavior included a fixed bonus applied to the value of the cued option. This bonus was positive meaning that participants were more likely to follow a partner’s advice when they made a fearful expression. Taken together, our results suggest that the subjective value of equally predictive social cues can be enhanced by additional emotional cues, but that this effect likely is variable among participants (see Fig 3). Nevertheless, we interpret our findings to demonstrate that emotional cues shape instrumental decision making similar to how they serve to transfer value in passive viewing tasks [11, 12].

Finally, we investigated how AQ scores related to performance on our study. We found no correlations between task performance (safe choices) and AQ scores irrespective of if participants were facing predictive partners or not. This was surprising as past research has indicated that people high in autistic traits may be impaired in tasks involving the integration of social cues [10, 13]. In one recent study [13], the reliability of the social cue was volatile, unlike in our study where different partners had different but fixed levels of volatility. In [10], both a volatile and a stable condition were used and correlations with participants’ AQ scores were found in both conditions, even if the correlations were smaller in the stable condition. This suggests that our findings cannot be explained by the stability of the partners. However, another difference between our study and previous ones is that we use an aversive decision making setting, where participants receive motivationally salient punishers (shocks) as feedback to their decisions. Past research, in non-social settings, has shown that persons with high-functioning autism spectrum disorder can perform better in risky decision making tasks by adopting safer and more risk averse strategies [39]. Therefore, a possible explanation for our findings might be that certain deficiencies associated with AQ scores are not as readily expressed in aversive settings. Consistent with this proposed explanation, we found a correlation between AQ scores and the difference in learning rates about the options and learning rates about the partners. Follow up analyses showed that this relationship appeared uniquely for participants in the instructed condition. This result was surprising and may indicate that participants with greater autistic traits regulate their attention to favor non-social information when they are informed that they will be in a situation where social information may play a role. This regulation of attention might compensate for the deficits in integrating social information they otherwise might have exhibited. While our sample size was large compared to that of previous studies investigating gaze cues and instrumental learning, our findings relating to AQ scores are nevertheless to be considered exploratory and should be replicated and extended in future investigations. In particular, it will be important to compare reward and punishment feedback conditions within-subjects to determine the role, if any, played by the type of feedback for social cue integration.

There are additional limitations and possible extensions to the present work that future studies should address. One concerns the relative anonymity of the partners. Ecological validity could be improved by giving participants more information about the partners, such as group belonging, status or trustworthiness, information that is typically available during real life interactions and that is known to affect the influence of social information. For example, people might be more inclined to use information from partners about whom they have more (positive) knowledge [40], hence it would be important to understand how this affects their learning. Further, by giving participants information about partners, it would be possible to better understand if and how such information shapes how participants use partner reliability in novel choice contexts. Another interesting extension of the current research is to increase realism by porting the task to a virtual reality setting. In such a setting it would be easy to create situations where participants interact with multiple social partners who could be controlled by other participants or confederates. Such a setting would come closer to modeling the richness of everyday interactions while still allowing for large degrees of experimental control [41]. Finally, in our experiment social partners were either fully predictive or fully random. While this design allowed us to clearly contrast the two and consider how learned information about social partner transferred between contexts, future work should explore how varying levels of partner reliability are learned and integrated.

Taken together, our findings demonstrate that people mix socially derived information with individual experiences to make decisions in aversive environments and are capable to track the value of multiple individuals across decision contexts. Using a reinforcement learning framework we show that participants cache social values and use these to inform their choices in novel context. The manner in which participants use previously learned social information likely reflects an ecologically valid risk-minimizing strategy. When facing uncertainty about what is the safest course of action, social advice can rapidly improve one’s chances of avoiding harmful consequences. Overreliance on social partners entails a risk of being deceived or misled if the source of advice is no longer valid and consequently diminishes one’s opportunities to learn about options for oneself. On the other hand, ignoring social information wastes accumulated knowledge and can overexpose oneself to dangers. Our findings indicate that people navigate this dilemma by opting for a middle-of-the-road strategy.

## Materials and methods

### Ethics statement

The experimental procedures were approved by the regional ethical committee at Karolinska Institutet (2012/340-31/4), and was carried out in accordance with the principles of the revised Helsinki Declaration. Written consent was obtained from all participants.

### Participants

We recruited 81 participants from the student population at Karolinska Institutet and from the local community. 40 participants were assigned to the naïve condition and the remainder to the instructed condition. Participants were give two cinema vouchers in exchange for their participation.

### Equipment and materials

The experiment was presented using PsychoPy [42]. The four faces used to represent the partners providing the gaze cues were taken from the Karolinska Directed Emotional Faces database (KDEF; [43]; Fig 1B). We used the neutral and fearful versions of each of the faces, and were edited in Adobe Photoshop to provide the gaze cues. The following KDEF image IDs were used in the study: AF01ANS, AF01NES, AF14ANS, AF14NES, AF26ANS, AF26NES, AF29ANS, AF29NES. Twenty-four fractal images were used as choice stimuli. All stimuli, fractals and faces, were presented in greyscale and modified to be isoluminant with the background color (RGB: 192 192 192).

Mild electric shocks, consisting of a single 100ms DC pulse, were administered using a Biopac STM200 module (Biopac Systems Inc.) applied to the lower forearm of participants’ dominant side. The strength of the electric shocks was individually calibrated so that participants experienced the shocks as being “unpleasant but not painful”.

### Experimental procedure

Participants entered the lab and were given general information about the experiment and consent forms to sign. They were the introduced to the shock delivery equipment and shock level was calibrated individually. Participants were informed during calibration that they would receive shocks based on their performance, but that they should expect around forty shocks due to the probabilistic nature of the reinforcement.

The main experiment consisted of a two-alternative forced choice task between two aversive options. Participants were told that the options differed in the likelihood of giving them an electric shock, but not what the objective probabilities were and had to learn these to perform optimally in the task. One option always terminated with shock if chosen with *P* = .8 and the other with *P* = .2.

The experiment was divided into twelve blocks of twelve trials. Each block featured novel options (fractal images). To prevent spatial decision strategies left/right position of options varied randomly between trials. Additionally, on each trial the face of a partner was shown. The identity of this partner was the same within a block. There were four unique partners in the experiment, meaning each participant met all partners in three separate blocks, therefore creating the opportunity for repeated encounters of a social partner in a new context. Two of the partners were *predictive* and two were *random*. Predictive partners *always* made a gaze cue towards the *bad* option. The direction of random partners’ gaze cues was determined on each trial with equal probability ensuring their cues had no predictive validity. Additionally, two of the partners, one *predictive* and one *random*, made their gaze cue with a *fearful* emotional expression, while the other two partners retained a *neutral* expression. All partners were encountered once before before participants re-encountered them.

Participants were additionally in one of two possible instruction conditions. In the *naïve* condition participants were only told that the face of a partner would be seen on each trial. In the *instructed* condition, in contrast, participants were additionally told that the some partners would have knowledge about what option was the safe one. Participants were not led to believe, however, that the social partner was being controlled by a confederate.

Each trial had the same structure, see also Fig 1A. First, a fixation cross was presented onscreen for 3s. After this a face of one of the four partners was displayed in the center of the screen. After 1.3s the partners gaze shifted to the left or right direction, thus providing a gaze cue. After 0.5s two fractals appears on each side of the partner, hence one fractal would appear in the cued direction and one in the non-cued direction. Participants were given free amount of time to choose, by pressing the left or right arrow keys, one of the fractals. Following their choice, the selected fractal was highlighted with a yellow frame and the non-selected fractal disappeared. The face of the partner remained onscreen. After 5.5s the trial terminated with either a shock or no feedback. The experiment consisted of 12 blocks of 12 trials each, resulting in 144 trials total.

At the end of the experiment, participants were asked to rank the partners by first selecting the partner they thought was most helpful, then selecting who they thought was the least helpful from the remaining three, and, lastly, selecting from the final two who was most helpful of those. Finally, participants filled in a 50 item Autism Questionnaire (AQ; [32]). Participants were then thanked, paid and debriefed.

### Computational models of behavior

To understand participants trial-by-trial choices we formulated several reinforcement learning models and compared their fit to participants’ data. Here we detail 14 different models (see Table 1). Some additional variations are reported in the S1 Text as explained below. We first give a general description of our modeling framework and then outline how each model differs from the others.

We assumed that participants learn the probabilities of options being safe, *p*_*i*_, *p*_*j*_, and the probability of each social partner giving good advice—signaling the safe option, *p*_*partner*_. We assumed that participants used these learned probabilities to calculate the expected value of each option as well as the expected value of the partner’s advice, by taking a shock as having reward value of −1 and the absence of shock reward value of 1. Hence the equation for expected values of partners’ advice and of each option is given by:

$$\begin{array}{c} EV_{x}=p_{x}\times 1+\left(1-p_{x}\right)\times -1 \end{array}$$ (1)

The expected values were combined such that the EV of the partners’ advice was combined with the EV of the option the partner was not looking at (i.e. signaling was safe by virtue of how the gaze cues were set-up in this experiment). Choices were generated with a softmax:

$$\begin{array}{c} Q_{x}=\{\begin{array}{cc} \omega EV_{x}+\left(1-\omega \right)EV_{partner} & \text{if}\;\text{x}\;\text{advised}\\ \omega EV_{x} & \text{otherwise} \end{array} \end{array}$$ (2)

$$\begin{array}{c} P_{i\;|\;i\;\text{advised}}=\frac{e^{Q_{i}/\beta }}{e^{Q_{i}/\beta }+e^{Q_{j}/\beta }} \end{array}$$ (3)

where *β* is an inverse gain parameter. Lower *β* implies more deterministic choices in favor of the option with the currently highest EV. In Eq (2) *ω* is a weighting parameter determining the relative influence of social advice versus self-experienced information about the options. As detailed below the different models differed in how *ω* was determined.

Updating followed a simple Rescorla-Wagner delta rule [44]. For options, *r* = 1 if the chosen option was safe and 0 otherwise:

$$\begin{array}{c} \delta =r_{t}-{\hat{p}}_{x,t} \end{array}$$ (4)

For learning about the partner, *r* depended on if the participant chose according to the partner’s advice or not, and ensures that the learning is congruent with the reinforcement received relative to the gaze direction of the partner:

$$\begin{array}{c} \delta_{partner}=\{\begin{array}{cc} r_{t}-{\hat{p}}_{partner,t} & \text{if}\;\text{advice}\;\text{followed}\\ \left(1-r_{t}\right)-{\hat{p}}_{partner,t} & \text{if}\;\text{advice}\;\text{not}\;\text{followed} \end{array} \end{array}$$ (5)

Finally, the chosen option and the partner’s estimated probabilities were updated:

$$\begin{array}{c} {\hat{p}}_{x,t+1}={\hat{p}}_{x,t}+\{\begin{array}{cc} \alpha_{+,x}\times \delta & \delta_{x}>0\\ \alpha_{-,x}\times \delta & \delta_{x}<0 \end{array} \end{array}$$ (6)

where *α* is the learning rate. We allowed the learning rate to vary between learning from experience and social learning, as well as for positive and negative prediction errors (*α*_+,*opt*_/*α*_−,*opt*_ and *α*_+,*partner*_/*α*_−,*partner*_ respectively), implying a total of four possible learning rate parameters. In the S1 Text we report additional comparisons based on models containing fewer learning rate parameters (see Table A in S1 Text)., however all these models exhibited worse fits to the data.

#### Option only

The option only model (**model 1**) was a null model that assumes that participants only learn about the options but not about the reliability of social partners’ advice. In this model, (*α*_+,*partner*_/*α*_−,*partner*_ were clamped to 0 and *ω* fixed to 0.5.

#### Gaze only

The gaze only model (**model 2**) was a null model that assumes that participants only learn about the reliability of social partners’ advice but not about the options. In this model, *α*_+,*opt*_/*α*_−,*opt*_ were clamped to 0 and *ω* fixed to 0.5.

#### Weak and strong transfer

Models that incorporated learning both about options and about social partners differed in how social information was cached and transferred between blocks. In our experiment, each block entailed novel choice options. In the *weak transfer* class of models (**models 3,5,7,9,11,13**), between blocks, *p*_*i*_, *p*_*j*_ are reset to their starting value of 0.5—implying *EV* = 0—since options are novel. Crucially, *p*_*partner*_ is cached, allowing information learned about the social partner’s usefulness to continue to influence decision making when facing a new, unknown environment.

By contrast, in the *strong transfer* class of models (**models 4,6,8,10,12,14**). *p*_*i*_, *p*_*j*_ are set to *p*_*partner*_ and 1 − *p*_*partner*_ on the first trial of each block, according to the advice given by the partner on that first trial. In environments where partners are continuously reliable (see Fig 1C, like that in our experiment, this algorithm efficiently scaffolds earlier learning.

#### Equal weighting

In **models 3-4** participants learned both about options and social partners. In these models expected values of options and of partners’ advice was assumed to be weighted equally, hence *ω* was fixed to 0.5.

#### Variable weighting

**Models 5-6** relaxed the assumption of equal weighting and allowed *ω* to vary freely.

#### Emotion weighting

**Models 7-8** tested the possibility that participants weighted the social partners’ advice differently if it came from a partner with a *fearful* expression compared to a partner with a *neutral* expression. To achieve this we introduced two weighting parameters *ω*_*f*_ and *ω*_*n*_ which took the place of *ω* in Eq (3) depending on the social partners’ emotional expression.

#### Emotion bonus

The emotion bonus models (**models 9-10**) tested another way partners’ emotional expressions might influence decision making. These models expanded on models **5-6** by adding a static bonus parameter, *θ* to the softmax, replacing Eq (2) with:

$$\begin{array}{c} Q_{x}=\{\begin{array}{cc} \omega EV_{x}+\left(1-\omega \right)EV_{partner}+\theta & \text{if}\;\text{x}\;\text{advised}\\ \omega EV_{x} & \text{otherwise} \end{array} \end{array}$$ (7)

*θ* could assign a positive or negative value to the option advised by partners who were making a *fearful* expression and took the value 0 for *neutral* partners.

#### Arbitration

The arbitration models (**models 11-12**) incorporated an alternative method of determining *ω* in Eq (2) by allowing it to vary on a trial-by-trial basis as suggested in recent work [18]. The key idea is that the weighting between different sources should be determined by their relative reliability. In this instance reliability was determined by comparing the absolute prediction errors from the chosen option (|*δ*|) and partner (|*δ*_*partner*_|) from the preceding trial. Arbitration was implemented as a softmax:

$$\begin{array}{c} \omega =\frac{e^{\left(1-|\delta |\right)}}{e^{\left(1-|\delta |\right)}+e^{\left((1-\right|\delta_{partner}\left|)+\gamma \right)}} \end{array}$$ (8)

Arbitration was biased by an additional parameter *γ*. This parameter reflected a bias towards the advice of the partner if *γ* < 0 and a bias towards the option if *γ* < 0. In the S1 Text we report variations of the arbitration models without the bias parameter as well as using a different arbitration scheme based on computing entropy rather than using absolute prediction errors [18]. These variations showed worse fit to our data (see Table B in S1 Text).

#### Arbitration emotion bonus

**Models 13-14** used the same emotion bonus parameter *θ* as in models **9-10** by using Eq (7) but determined *ω* as in models **11-12**.

#### Hierarchical Gaussian Filter

In the S1 Text we additionally report on fitting the Hierarchical Gaussian Filter [45] to our data, as this model has previously been used to model data from experiments similar to ours [13].

### Model fitting and comparison

All our computational models were implemented in the Stan probabilistic programming language and fit with MCMC sampling using the NUTS sampling algorithm [46]. All parameters were fit hierarchically to each participant as deviations from an estimated population average. Parameters were fit in logit space and then back-transformed to their native space. Priors for all parameters were set as *Normal(0,1)* except for population-level learning rates were weakly informative *Normal(0,0.5)* were used to assist convergence.

Learning rate (*α*) and weighting (*ω*) parameters were constrained in the interval [0, 1] and the temperature parameter (*β*) constrained in the interval (0,2]. All *p*’s were initially set to 0.5. The emotion bonus (*θ*) and arbitration bias (*γ*) parameters were constrained to the interval [-1, 1].

Model comparison was performed using leave-one-out cross-validation (LOO-CV) by estimating the pointwise out-of-sample prediction accuracy from the log-likelihood evaluated using the full posterior of the model [33]. Since individual observations are not independent in trial-by-trial computational models, we follow [47] and use the pointwise log-likelihood summed to the participant level as an input to the leave-one-out cross-validation procedure. See Table 2 for full model comparison.

We also simulated data from a subset of our candidate models (models **1-3, 5, 7, 9, 11, 13**) and fitted the models to the simulated data. The resulting confusion matrices are reported in the S1 Text (Fig A and Fig B). Generally, models with emotion expression components distinguish themselves well compared to other models. However, models **9** and **13** produce very similar data patterns to each other, which is also reflected in their fit similarity to our data (see Table 2).

### Statistical analysis

All analyses were performed in the R statistical language using the *brms* package [48]. Where appropriate we analyzed the data using Bayesian multi-level regression including varying intercepts and slopes by participant and correlations between intercept and slopes. All categorical regressors were deviation coded (-0.5/0.5) and all continuous regressors were standardized. In addition to the parameter estimate, its standard error and 95% credible intervals we also report the *probability of difference* (*pd*) [49]. The quantity *pd* is the proportion of the posterior distribution of the parameter that has the same sign as the parameter itself.

## Supporting information

S1 Text Supplementary methods and results. (PDF)
