# Supplementary material for: Integration of social cues and individual experiences during instrumental avoidance learning
Source: PLoS Comput Biol. 2020 Sep 8;16(9):e1008163. doi: 10.1371/journal.pcbi.1008163 (PMC7500672; doi:10.1371/journal.pcbi.1008163)
Supplement: S1 Text — (PDF) [file pcbi.1008163.s001.pdf]

SI Text for Integration of social cues and individual experiences during instrumental avoidance learning

Philip Pärnamets & Andreas Olsson

Supplementary Methods

Model comparisons with fewer learning rates

We fit models **1-6** with fewer learning rates by either forcing $\alpha_{+,opt}$ to be equal to $\alpha_{-,opt}$ or $\alpha_{+,partner}$ to be equal $\alpha_{-,partner}$ or both. As seen in Table A below, these alternatives consistently provided worse fits to our data.

Table A: Comparisons of **models 1-6** with different amounts of learning rates. LR = number of option learning rates. GLR = number of partner learning rates. Models in bold are the best of each type.

Model #	Learning rates	elpd_diff	se_diff	elpd_loo	se_elpd_loo	p_loo	se_p_loo
**5**	**2LR 2GLR**	0.00	0.00	-4172.40	165.51	248.78	7.03
**3**	**2LR 2GLR**	-66.31	16.42	-4238.72	160.93	245.42	8.31
5	2LR 1GLR	-121.58	27.75	-4293.99	170.24	225.71	7.39
3	2LR 1GLR	-138.90	27.10	-4311.30	168.68	212.60	7.23
**6**	**2LR 2GLR**	-146.08	27.37	-4318.49	160.96	297.55	9.47
**4**	**2LR 2GLR**	-167.86	27.81	-4340.26	165.06	270.24	8.17
5	1LR 2GLR	-203.98	37.89	-4376.39	166.07	243.85	8.97
6	2LR 1GLR	-224.84	31.76	-4397.25	168.18	236.70	7.89
4	2LR 1GLR	-248.51	35.46	-4420.91	168.70	218.68	6.87
3	1LR 2GLR	-289.42	44.63	-4461.82	160.35	238.77	10.54
6	1LR 2GLR	-315.23	45.71	-4487.63	160.45	304.50	11.17
5	1LR 1GLR	-327.01	46.31	-4499.42	171.96	212.01	6.94
3	1LR 1GLR	-337.58	46.03	-4509.99	171.59	190.24	6.29
4	1LR 2GLR	-340.30	47.79	-4512.71	165.45	261.91	10.33
6	1LR 1GLR	-418.22	46.77	-4590.62	167.66	232.26	7.53
4	1LR 1GLR	-442.77	50.29	-4615.17	169.90	205.03	7.21
**1**	**2LR**	-461.17	52.79	-4633.57	178.26	155.05	5.32
1	1LR	-730.05	67.30	-4902.46	179.78	118.07	5.77
**2**	**2LR**	-2047.01	137.13	-6219.41	92.31	118.03	7.24
2	1LR	-2153.30	129.08	-6325.70	87.17	111.79	7.46

Model comparisons of alternative arbitration models

Four additional arbitration models were considered detailed below, and full comparison between all models is shown in Table B, below.

The first was a simplified version of model **11**, which didn't have the γ parameter biasing arbitration. In other words, the equation determining weighting between option values and the partner value (Eq 7 main text) was simply:

$$\omega = \frac{e^{(1-|\delta|)}}{e^{(1-|\delta|)} + e^{((1-|\delta_{partner}|))}} \tag{8}$$

The second, third and fourth models considered implemented arbitration based on entropy rather than prediction errors [Charpentier et al., 2020]. Entropy was calculated as Shannon entropy as in Eqs 9 and 10. One model featured only entropy based arbitration, as in Eq 11 below. The next also included a fixed bias term (γ), analogous to model **11**, as seen in equation (12) below. The final model additionally has a term for biasing decisions when partners' made fearful expressions, analogous to model **13**.

$$H_{opt} = \sum p_{opt} * \log_2 p_{opt} \tag{9}$$

$$H_{part.} = p_{partner} * \log_2 p_{partner} \tag{10}$$

$$\omega = \frac{e^{(1-H_{opt})}}{e^{(1-H_{opt})} + e^{(1-(H_{part.}/0.5))}} \tag{11}$$

$$\omega = \frac{e^{(1-H_{opt})}}{e^{(1-H_{opt})} + e^{(1-(H_{part.}/0.5)+\gamma)}} \tag{12}$$

Table B: Comparisons of all arbitration models considered. Ent. = model with arbitration based on entropy only. Ent. γ = model with arbitration based on entropy with additional bias term. Ent. γ θ = model entropy arbitration with additional bias term and emotion bias. PE = model with arbitration based on prediction errors only.

Model	elpd_diff	se_diff	elpd_loo	se_elpd_loo	p_loo	se_p_loo
13	0.00	0.00	-4155.14	165.89	282.51	8.32
Ent. γ θ	-19.89	9.11	-4175.04	165.05	293.55	9.14
11	-32.45	11.04	-4187.59	164.61	246.28	7.53
Ent. γ	-53.22	13.49	-4208.36	164.47	254.54	8.39
PE	-67.67	14.33	-4222.81	161.71	248.50	8.42
Ent.	-103.62	17.12	-4258.76	161.37	255.83	8.72
14	-110.68	22.91	-4265.83	164.07	326.08	10.61
12	-150.44	26.91	-4305.58	162.24	298.84	10.10

Hierarchical Gaussian Filter model

We additionally explored an alternative modeling framework based on the Hierarchical Gaussian Filter (HGF) [Mathys et al., 2014], which has been used to explore data and test hypotheses on autism spectrum related differences in previous research [Sevgi et al., 2020]. We implemented the HGF in Stan [Carpenter et al., 2017], based on open code from Sevgi et al. [2020], using an implementation that estimated parameters hierarchically. We additionally modified the implementation to estimate option probabilities separately instead of jointly to make the model more comparable to our other models. Due to problems with acheiving model convergence when estimating the model using MCMC the model was instaed fit using maximum likelihood estimation using the L-BFGS-S optmization routine available in Stan.

We compared the per participant log-likelihood esimates of the HGF with the average posterior log-likelihood from model **9** by first calculating the Akaike Information Criteria (AIC) and then computing per participant AIC weights (AIC_w) [Wagenmakers and Farrell, 2004]. AIC weights can be interpreted as the strength of evidence for favoring one model over the other. Model comparison strongly favored model **9**, with it being the preferred model for 80 out of 81 participants. Median AIC_w for model **9** was 1.0 and average AIC_w was 0.989.

Simulations of model recovery

We simulated date from a subset of our candidate models (models **1-3, 5, 7, 9, 11, 13**) and fitted the models to the simulated data. For each model in the list above, we simulated response data for 80 participants, drawn from a parameter distrubion as detailed below. Each simulated dataset was then fit to each of the models under consideration. We repeated this process 250 times for each model. We used Stan's built in optimization engine (L-BFGS algorithm) to obtain maximum likelihood fits of each model to each simulated dataset and compared fits using Akaike weights [Wagenmakers and Farrell, 2004]. In a given simulated dataset, we selected a model as best fitting in that stimulation run if it was indicated as best fitting for most participants in the simulated dataset.

For all simulations parameters were drawn from the following distributions, where appropriate:

$\alpha_{+/-,partner,option} \propto \text{Normal}(0.5, 0.1)$

$\beta \propto \text{Normal}(0.2, 0.05)$

$\omega \propto \text{Unif}(0.7, 0.8)|\text{Unif}(0.2, 0.3)$

$\omega_f \propto \text{Unif}(0.025, 0.125)$

$\theta \propto \text{Normal}(0.4, 0.1)$

$\gamma \propto \text{Normal}(0.6, 0.05)|\text{Normal}(-0.6, 0.05)$

To assess to how often each model was best fit to datasets generated according to the specifications of the different models we compiled confusion matrices. We first compared the models excluding the two arbitration models (**11, 13**). Models with emotional components are especially well identified. Fig B plots the confusion matrix for all models considered. Note that in Fig B, models **9** and **13** are strongly confused, which is also mirrored in the fits to the empirical data.

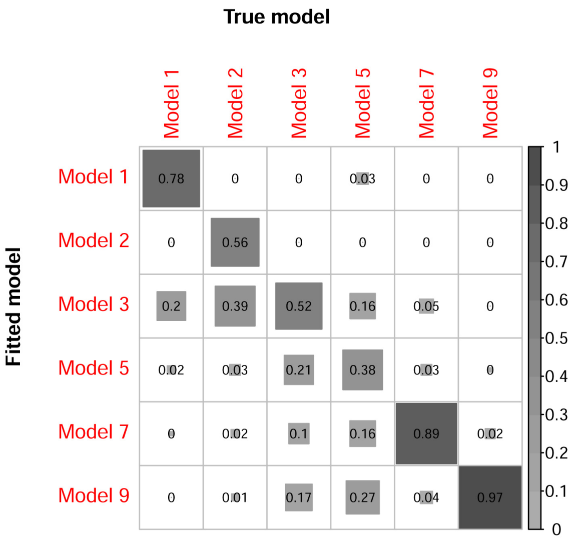

Figure A: Confusion matrix showing the proportion (of 250 simulation runs) each model was considered best fitting to a dataset generated according to the specification of each of the models indicated.

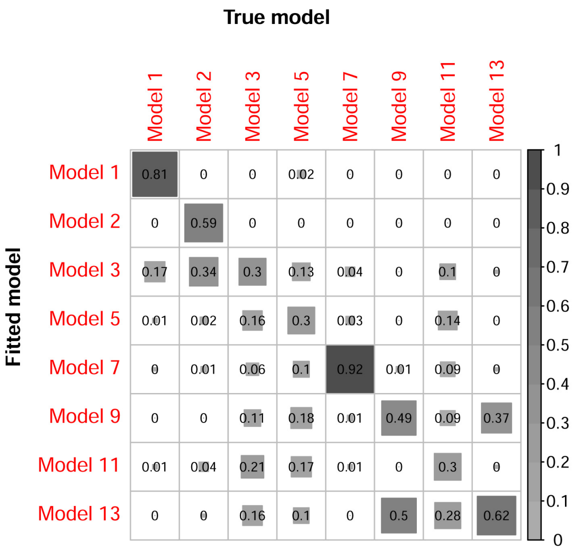

Figure B: Confusion matrix showing the proportion (of 250 simulation runs) each model was considered best fitting to a dataset generated according to the specification of each of the models indicated.

Supplementary Results

Effects of parameter combinations on simulation results

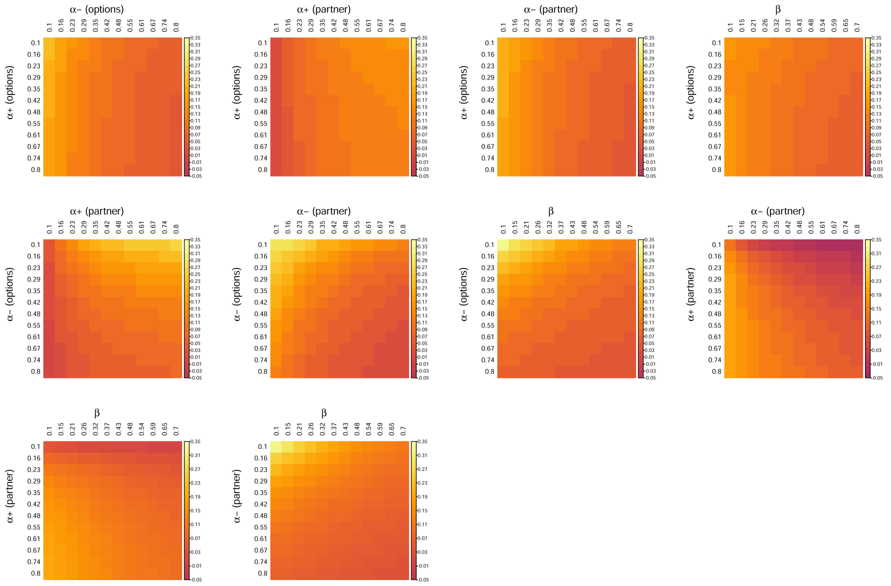

Figure C: Differences in safe choices for all parameter combinations. Average difference in proportion safe choices between weak and strong transfer models for different parameter combinations when social partner reverses their advice.

Correlations between option-partner learning rates and AQ

We explored if the correlation between the difference in average option learning rates and average partner learning rates on the one hand and AQ scores on the others was robust to other specifications of the learning model. To do so we assessed the same correlation in the two next best fitting models **5** and **13**. We found for the model **5** an almost identical correlation (within rounding error) (robust correlation, $r = 0.19$, $SE = 0.11$, 95% CrI = [-0.04, 0.39], $pd = 0.950$). For the model **13** (*arbitration model with emotion bonus*) the correlation was weaker but consistent with the other two results (robust correlation, $r = 0.11$, $SE = 0.11$, 95% CrI = [-0.11, 0.32], $pd = 0.838$).

Differences of model parameters on instruction condition

If instructions attuned participants to the role of the social partner it might be possible to see differences in learning rates or in weighting of social information in the decision function. We tested these possibilities in turn. We

first took each participant's average learning rate to social partners, and compared participants by instruction condition. We found no differences (Bayesian t-test, b = -0.010, SE = 0.015, 95% CrI = [-0.039, 0.019], pd = 0.751). Second, we compared participants' ω parameter which captured the weighting between option and social information in during decision-making. Again we found no differences by instruction condition (Bayesian t-test, b = 0.012, SE = 0.029, 95% CrI = [-0.043, 0.069], pd = 0.659). A final possibility we investigated, in light of interaction between emotional expression and instruction condition, was if there was a tendency for θ to be higher in the instructed condition. We found no reliable difference even if it was directionally consistent with this conjecture (Bayesian t-test, b = 0.010, SE = 0.008, 95% CrI = [-0.006, 0.025], pd = 0.903).

Additional analyses partner ratings

We tested if participants who were better fit by a strong transfer **model 10** showed more preference for predictive partners compared to those who were better fit by a weak transfer **model 9** . To assess this we extracted the average posterior log-likelihood per participant for the two models. We then scored participants rankings, such that placing a predictive partner in places 1 or 2 earned a one point for each partner and placing a random partner in places 3 or 4 also earned one point. Participants could thus be scored as ranking 0, 2 or 4 partners correctly and we divided the score measure by 2 so that it would range 0-2. Finally, participants scores were regressed on to the per participant log-likelihood difference (range -3.6 to 8.4) between the two models, using an ordinal probit regression. The analysis did not indicate a clear effect of log-likelihood difference on the ranking score (b = -0.044, SE = 0.055, 95% CrI = [-0.150, 0.064], pd = 0.782).

Hierarchical Gaussian Filter

We investigated if some of the fitted parameters of the HGF model correlated with participants' AQ scores. We first investigated the κ_{option} parameter, which is a phasic learning rate parameter regulating the coupling between levels in the HGF model. In Sevgi et al. [2020] differences are reported on that parameter for low AQ participants depending on if they (between-subjects, N=10 per group) first experience the social partner giving good advice or bad advice. No differences are reported contrasting high and low AQ scorers. While there is no analogy in our experiment to the treatment condition in their study, we reasoned that we could reasonably explore if there are correlations in the κ_{option} parameter with AQ scores in our sample. Our analyses indicated a small and somewhat probable correlation (robust correlation, r = 0.14, SE = 0.10, 95% CrI = [-0.064, 0.33], pd = 0.913). We therefore next tested if differences in κ_{option} and κ_{gaze} correlated with AQ

scores, analogous to the analyses we reported above for learning rates in our RW models. Here we found no strong indication of a correlation (robust correlation, r = 0.098, SE = 0.10, 95% CrI = [-0.099, 0.292], pd = 0.835). We conclude that even if these findings are uncertain, they do not directly contradict previous research.

References

B. Carpenter, A. Gelman, M. D. Hoffman, D. Lee, B. Goodrich, M. Betancourt, M. Brubaker, J. Guo, P. Li, and A. Riddell. Stan: A probabilistic programming language. *Journal of statistical software*, 76(1), 2017.

C. J. Charpentier, K. Iigaya, and J. P. O'Doherty. A neuro-computational account of arbitration between choice imitation and goal emulation during human observational learning. *Neuron*, 2020.

C. D. Mathys, E. I. Lomakina, J. Daunizeau, S. Iglesias, K. H. Brodersen, K. J. Friston, and K. E. Stephan. Uncertainty in perception and the hierarchical gaussian filter. *Frontiers in human neuroscience*, 8:825, 2014.

M. Sevgi, A. O. Diaconescu, L. Henco, M. Tittgemeyer, and L. Schilbach. Social bayes: Using bayesian modeling to study autistic trait–related differences in social cognition. *Biological Psychiatry*, 87(2):185–193, 2020.

E.-J. Wagenmakers and S. Farrell. Aic model selection using akaike weights. *Psychonomic bulletin & review*, 11(1):192–196, 2004.
